# The characteristics and outcomes of COVID‐19 among diabetic patients in Wad‐Medani isolation center from September to December 2020: A cross‐sectional study

**DOI:** 10.1002/hsr2.889

**Published:** 2022-10-31

**Authors:** Maali Mustafa, Alaaeldeen Attaallah, Osman Amir, Moawia Elbalal, Abdallah Abd AL‐Kareem Gibriel

**Affiliations:** ^1^ Department of Medicine Wad Medani Teaching Hospital Wad Medani Sudan; ^2^ Internal Medicine Faculty of Dentistry, University of Gezira Wad Medani Sudan; ^3^ Department of Hematology Faculty of Medical Laboratory Sciences, Al‐Neelain University Khartoum Sudan; ^4^ Internal Medicine Faculty of Medicine, University of Gezira Wad Medani Sudan

**Keywords:** COVID‐19, diabetes, inflammation, Sudanese

## Abstract

**Background and Aims:**

Corona virus disease‐19 (COVID‐19) is a recently discovered infection that transmitted briskly worldwide. In this disease (COVID‐19), it was discovered that several disorders, such diabetes, increased the severity and fatality rate. Until now, studies investigating the correlation between diabetes and COVID‐19 in Sudan have not yet been conducted. Thus we aimed to evaluated the characteristics and outcomes of COVID‐19 among diabetic patients

**Methods:**

A prospective study included 70 diabetic patients with COVID‐19 in Wad‐Medani Isolation Center from September to December 2020. Data concerning demographics and clinical characteristics, as well as outcomes were collected.

**Results:**

Out of 70 patients, 46 (66%) were men and 24 (34%) were women; the average age was 63 ± 12 years. In diabetes mellitus (DM) types, 69 (98.6%) patient were T2DM. The average of DM duration was 10 ± 6.2 years. Insulin was the major DM medication used by more one‐half of study patients (*n* = 37; 52.9%). Newly discovered DM after COVD‐19 infection was encountered in 5 (7.1%) patients. Most of the study subjects (*n* = 44; 63%) had moderately severe form of disease. Hypertension was the commonest comorbid in 29 (41.4%) patients. The intensive care unit admission rate among our study group was 10% (*n* = 7). The mortality rate among our study patients was found to be 11.4% (*n* = 8). Dead patients were significantly had high HbA1c levels (11.6 ± 7% vs. 8.8 ± 5%; *p* = 0.001). Additionally, all individuals with a severe COVID‐19 illness (*n* = 6; 100%) were dead comparing to no patient died with mild covid illness and 4.5% patients with moderately severe infection (*p* < 0.001).

**Conclusion:**

The majority of COVID‐19 diabetic patients were males and older in age. Most of the patients presented with moderate severity and moderately uncontrolled DM. Hypertension was the major comorbidity. The mortality rate was as high as 11.4% and associated with high HbA1c levels and severe form of COVID‐19 as well.

## 1 INTRODUCTION

Initially identified as a viral pneumonia outbreak in Wuhan, China, in December 2019, the corona virus disease (corona virus disease‐19 [COVID‐19]) has quickly transmitted and presented a public health issue.^1^, ^2^ As February 25, 2020, the World Health Organization (WHO) proclaimed COVID‐19 to be a pandemic and 81,109 laboratory confirmed subjects had been registered globally.^3^ Until the time of study writing (August, 2022), over 574 million infected subjects and over 6.3 million deaths have been stated internationally.^4^

The first COVID‐19 case in Sudan was stated on March 13, 2020^5^ and there were more than 63,136 confirmed cases with over 4961 deaths as of August, 2022.^6^

The COVID‐19 clinical features could be ranged from asymptomatic (17.9%–33.3%) or flu‐like presentations to serious condition of acute respiratory failure demanding mechanical ventilation, sepsis and several organ failures. According to evaluations, the mean incubation period is 5.1 days, and most of cases could show symptoms as early as 11.5 days.^7^

Diabetes mellitus (DM) is a metabolic disorder with increasing severity over the previous two decades, it's also act as trigger increasing respiratory tract infections.^8^ Several works have demonstrated that COVID‐19 is correlated to increasing blood glucose levels, especially in elderly DM cases.^9^ In one study in Wuhan included 1561 COVID‐19 cases, DM was significantly increased the likehood of intensive care unit (ICU) admission and mortality.^10^ Also, in British cohort that enrolled 5693 cases, the hazard of fatality was higher among diabetics (*p* < 0.05), but, the poor outcomes are due to diabetes per se or associated comorbidities still not fully clarified.^11^

Up to now, information concerning the clinical characteristics and the outcomes of diabetic COVID‐19 subjects was scarce particularly in Sudan. Therefore, this work aimed to evaluate characteristics and outcomes of diabetic COVID‐19 Sudanese cases.

## 2 MATERIALS AND METHODS

### 2.1 Study design

This is a prospective cross‐sectional study conducted in Wad‐Medani Isolation Center in Gezira State during the period from September to December 2020.

### 2.2 Study setting

Wad‐Medani Isolation Center is one of the major COVID‐19 referral centers found in Gezira State that receives COVID‐19 cases from the Central Sudan states. Inside this center, there are 65 beds for mild to moderate patients as 45 beds in general ward as well as 20 beds in high dependency unit. Moreover, this center contains pharmacy, two dialysis units, and a 24‐h laboratory.

### 2.3 Study participants

In this is study a total of 70 adult known or newly diagnosed diabetic patients with COVID‐19 diagnosed by defined reverse‐transcriptase polymerase chain reaction test by nasopharyngeal swab were recruited. Nondiabetic patients were excluded

The diagnosis of known or newly diagnosed DM was performed by oral glucose tolerance test after 75 g anhydrous glucose administration as follow: fasting palms glucose more than 126 mg/dl, and/or 2‐h postprandial glucose more than 199 mg/dl, and/or HbA1c more than 6.5%.

### 2.4 Data collection

The COVID‐19 case report form was created to collect primary clinical data concerning demographics, medical history, clinical symptoms, laboratory findings, and clinical outcomes. Age, gender, DM type, DM duration, use of DM drugs, HbA1c level, co‐morbidities, COVID‐19 severity, length of stay in the ICU, and patient outcome after 3‐month follow‐up (normally discharged or deceased) were the data used in the current study.

The severity of COVID‐19 was categorized into three groups including mild, moderate, as well as severe, according to the Sudanese Federal Ministry of Health's General Directorate of Health Emergencies and Epidemic Control.

The ICU admission criteria included; sever pneumonia CURB > 3, coma, post arrest, Pao_2_/Fio_2_ < 250, mechanical ventilation, hypotension or hemodynamic unstability.

### 2.5 Data analysis

Data analysis was performed by Statistical Package for Social Sciences program (SPSS, Version 21). Categorical variables were presented in frequencies with percentages as well as continuous variables in means with standard deviations. Kolmogorov–Smirnov normality test was applied to study variables. Chi‐Square test was used in bivariate analyses to determine the factors associated with the outcomes. All *p* values were considered as significant at level 0.05 (two‐sided).

### 2.6 Ethical approval

Ethical approval obtained from National Research Ethics‐review Committee in Sudan Medical Specialization Board. Patients' informed permission and approval from hospital management were also acquired. In attempt to preserve the patient's identity, data utilized privately by using numbers rather than names. The research reports did not mention any specific participants. Only research staff was aware of the patients' identities.

## 3 RESULTS

Among 70 COVID‐19 diabetic patients, 46 (66%) were men and 24 (34%) were women, their average age was 63 ± 12 years and the majority of them 37 (52.9%) aged above 60 years. DM duration was 10 ± 6.2 years. The majority of the patients 37 (52.9%) used insulin as antidiabetic. The mean of glycosylated was found to be 9.2 ± 2.4%. Newly discovered DM after COVD‐19 was found in 5 (7.1%) patients. About two‐third of patients (*n* = 44; 63%) presented with moderate COVID‐19 infection. The median of hospital stay was found to be 9 days ranged from 1 to 28 days. The characteristics of patients showed in Table 1.

**Table 1:** Characteristics of diabetic COVID‐19 patients (*N* = 70)

	*N*	%
Age (years); mean ± *SD*	63 ± 12	
<40	6	8.5
40–60	27	38.6
>60	37	52.9
Gender (male)	46	66
DM duration (years); mean ± *SD*	10 ± 6.2	
<5	17	24.3
5–10	30	42.9
>10	23	32.9
DM medications		
Insulin	37	52.9
OADA	22	31.4
Metformin	8	11.4
Insulin + OADA	2	2.9
Diet control	1	1.4
Hypertension	29	41.4
CVD	20	28.6
Pulmonary disease	4	5.7
Renal disease	3	4.3
Newly diagnosed DM after COVID‐19	5	7.1
HBA1c (%); mean ± *SD*	9.2 ± 2.4	
<7	7	10.0
7–9	38	54.3
>9	25	35.7
COVID‐19 severity		
Mild	20	28.6
Moderate	44	62.9
Severe	6	8.6
ICU admission	7	10
Hospital stay (days); average (mini–max)	9 (1–28)	

Abbreviations: COVID‐19, corona virus disease‐19; CVD, cardiovascular disease; DM, diabetes mellitus; HbA1c, glycosylated hemoglobin; ICU, intensive care unit; OADA, oral antidiabetic agent.

**Figure 1:**
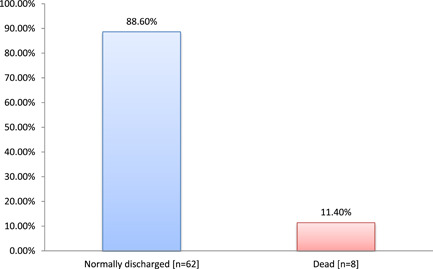
The outcomes of COVID‐19 diabetic patients (*N* = 70). COVID‐19, corona virus disease‐19

In outcomes, 8 (11.4%) patients were dead and remaining 62 (88.6%) patients were discharged as illustrated in Figure 1.

As revealed in Table 2, deceased patients were significantly had higher HbA1c levels comparing to discharged patients (11.6 ± 7% vs. 8.8 ± 5%; *p* = 0.002). All patients with severe SARS‐CoV‐2 (severe acute respiratory syndrome corona virus 2) illness (*n* = 6; 100%) were dead comparing to no patients died  with mild covid 19 illness  and 4.5% patients with moderately severe infection (*p* = 0.000). Nonsurvivors were more tended to be admitted to ICU (*n* = 7; 100%) incontrast to discharged patients (*p* < 0.001).

**Table 2:** Characteristics of diabetic COVID‐19 patients with diabetes by their outcome

	Outcome		
	Discharged; *n* (%)	Dead; *n* (%)	*p* value
Age (years); mean ± *SD*	62.5 ± 12	67 ± 4	
<40	6 (100)	0 (0)	0.220^a^
40–60	26 (96.3)	1 (3.7)	
>60	30 (81.1)	7 (18.9)	
Gender			
Male	41 (89.1)	5 (10.9)	0.561^a^
Female	21 (87.5)	3 (12.5)	
DM duration (years); mean ± *SD*	10 ± 6	9.8 ± 6.2	
<5	14 (82.4)	3 (17.6)	0.664^a^
5–10	27 (90)	3 (10)	
>10	21 (91.3)	2 (8.7)	
DM medications			
Insulin	32 (86.5)	5 (13.5)	0.371^a^
OADA	21 (95.5)	1 (4.5)	
Insulin + OADA	1 (50)	1 (50)	
Diet control	1 (100)	0 (0)	
Metformin	7 (87.5)	1 (12.5)	
Hypertension	26 (41.9)	3 (37.5)	0.563
CVD	18 (29)	2 (25)	0.588
Pulmonary disease	3 (4.8)	1 (12.5)	0.392
Renal disease	2 (25)	1 (12.5)	0.309
HBA1c (%); mean ± *SD*	8.8 ± 5	11.6 ± 7	
<7	7 (100)	0 (0)	**0.002** a, b
7–9	37 (97.4)	1 (2.6)	
>9	18 (72)	7 (28)	
COVID‐19 severity			
Mild	20 (100)	0 (0)	**<0.001** a, b
Moderate	42 (95.5)	2 (4.5)	
Severe	0 (0)	6 (100)	
ICU admission			
Yes	0 (0)	7 (100)	**<0.001** a, b
No	62 (98.4)	1 (1.6)	
Hospital stay (days); mean ± *SD*	9.5 ± 5	10.6 ± 7	0.596^a^

*Note*: Bold values are highly significant.

Abbreviations: COVID‐19, corona virus disease‐19; CVD, cardiovascular disease; DM, diabetes mellitus; HbA1c, glycosylated hemoglobin; ICU, intensive care unit; OADA, oral antidiabetic agent.

^a^ Chi‐square test.

b Significant (<0.05).

## 4 DISCUSSION

This study aimed to evaluate the characteristics and outcomes of SARS‐CoV‐2 among 70 diabetic patients in Wad‐Medani Isolation Center, Gezira state, Sudan.

Among this study's subjects, DM patients with COVID‐19 were commonly males (*n* = 46; 66%) with a male to female ratio of (1.9:1). Similar results were reported by Chinese study of Li et al.^8^ who reported 57.1% of admitted diabetic patients were males. Also, Williamson et al.^11^ and Alamin et al.^12^ noticed that 52% and 62.5% of diabetic COVID‐19 patients were males, respectively.

The average age of our patients was found to be 63 ± 12 years and the majority of them 37 (52.9%) aged above 60 years. Similar results were reported by Alamin et al.,^12^ Bode et al.^13^ and The Philippine CORONA Study Group^14^ those stated that COVID‐19 was common among older diabetic patients (>60 years). In the study of Williamson et al.,^11^ patients with diabetes were found to be 10 years older than non‐DM cases.

Insulin was the major DM medication used by more than one‐half of our study patients (*n* = 37; 52.9%). This result is attributable to the advantages of anti‐inflammatory properties of insulin, that are associated with enhanced immune mediators and the reduction of proinflammatory cytokines, in addition to favorable glycemic control achieved by insulin that illustrated by Sardu et al.^15^ who studies 25 diabetic COVID‐19 patients and found the average of the blood glucose during hospitalization was 10.65 ± 0.84 mmol/L in noninsulin‐treated compared to 7.69 ± 1.85 mmol/L in insulin‐treated groups (*p* < 0.001).

Despite the crucial role of metformin in suppression of SARS‐CoV‐2 pathogenicity by phosphorylation of ACE2 receptors (specific SARS‐CoV‐2 receptors) and suppression of mammalian target of rapamycin,^16^ only 8 (11.4%) patients in the present study have received metformin.

The newly diagnosed DM after COVD‐19 infection was encountered in 5 (7.1%) patients. The new onset DM after COVID‐19 could be developed by the direct beta cell pancreatic injury through ACE‐2 receptors expressed in the pancreatic cells.^17^ Our findings were comparable to Wang et al.^17^ in China who found that 9.6% of admitted COVID‐19 patients in Wuhan developed DM after COVID‐19. In contrast, Li et al.^8^ observed that among 453 SARS‐CoV‐2 patients, the frequency of newly diagnosed diabetes was 20.7%.

Diabetes increases the likelihood of acquiring COVID‐19 severe ilness and critical manifestations. Moreover, frailty and elevated inflammatory predictors were reported in diabetic subject in pandemic era.^18^ In the present study most of the study subjects (71.5%) had moderate‐severe form of disease. Same results were reported by Guozhen et al.^19^ who found that most of DM patients had moderate to severe SARS‐CoV‐2 disease.

In the current study hypertension was the commonest comorbidity in 29 (41.4%) patients. Our results were supported by Alamin A et al.^12^ who stated that hypertension was the major chronic disease in 47.1% of diabetic COVID‐19 patients. Since SARS‐CoV‐2 binds to ACE2 to enter target cells, it has been hypothesized that the widespread use of ACE inhibitors may be to accountable for the infection's high prevalence. Lung, heart, liver, kidney, ileum, and brain all express ACE2, which are physiologically engaged in anti‐inflammatory reactions.^11^ It was believed that ACE inhibitors and angiotensin receptor blockers might accelerate target organ infection and exacerbate the course of the disease since experimental data indicates that these medications boost the expression of ACE2.^11^

Diabetes patients with COVID‐19 had a greater likelihood of being admitted to the ICU. In the present study, 10% of the study group (*n* = 7) were admitted to the ICU. Correspondingly, in the study of Wang et al.,^20^ 8.2% of patients required intensive care.

In the present study, the mortality rate among our study patients was found to be 11.4% (*n* = 8). Several mechanisms have been stated for explaining the death among diabetic COVID‐19 patients included those specifically correlated to high blood sugar, resulting imbalances, and diabetes‐related comorbidities/complications.^21^ Our mortality rate was comparable to other previous studies such as Guozhen et al,^19^ Guo et al.,^22^ Wang et al.,^23^ and Wu et al.^24^ where mortality rates were 14.5%, 16%, 10.8%, and 7.3%, respectively. However, higher findings (35.5%) was reported by Onder et al.^25^

In attempt to detect the factors influencing fatality in diabetic SARS‐CoV‐2 patients, poor glycemic control was found to be a significant factor correlated with mortality among those patients as nonsurvived patients were significantly had high HbA1c levels compared to normally discharged patients (11.6 ± 7% vs. 8.8 ± 5%; *p* = 0.001). It is still unclear whether having poor glycemic control renders COVID‐19 individuals at risk for the severe illness, in Giuseppe et al.^26^ study the patients with well‐controlled blood glucose levels had lesser mortality rates compared to poorly controlled blood glucose levels counterparts (1.1% vs. 11.0%; *p* = 0.001). Results from a different research also revealed that individuals with T1DM or T2DM who had HbA1c levels greater than 86 mmol/mol (10%) had a higher risk of death than those with HbA1c levels lower than 48 mmol/mol (6.5%).^27^ In contrast, the CORONADO study found no significant correlation of HbA1c levels with death and MV in diabetic patients diagnosed with COVID‐19.^28^

COVID‐19 severity was another risk factor of mortality encountered in this study and all dead patients had severe COVID‐19 infection (*n* = 6; 100%) compared to no patient with mild and 4.5% patients with moderately severe infection (*p* = 0.000). Consistently, Wang et al.,^29^ Zheng et al.,^30^ and Tian et al.^31^ demonstrated that DM patients with severe SARS‐CoV‐2 infection had a greater odds of death more than non‐severe patients, unlike Yang et al.^32^

Finally, adherence to COVID‐19 prevention protocols by WHO and CDC particularly among patients with DM is recommended. Furthermore, it is highly suggested that diabetic COVID‐19 individuals to achieve optimal glucose control. Additionally, more comprehensive researches are needed to clarify certain unclear postulates about the relationship between DM and COVID‐19 illness.

The limitations of the current study could summarized in following; first, the cross‐sectional design that could not allow to us to generalize this findings, and secondly, this study only included diabetic patients thus further studies compared both diabetic and nondiabetic patients are required.

## 5 CONCLUSION

This study concluded that, diabetic patients with COVID‐19 were mainly males and older in age. Most of the patient presented with moderate severity and moderately uncontrolled DM. hypertension was the major comorbidity. The mortality rate was as high as 11.4% and associated with high HbA1c levels and severe form of COVID‐19.

## AUTHOR CONTRIBUTIONS

**Maali Mustafa**: Conceptualization; investigation; methodology; project administration; resources; writing – original draft. **Alaaeldeen Attaallah**: Investigation; methodology; resources; writing – review & editing. **Osman Amir**: Data curation; formal analysis; methodology; writing – original draft. **Moawia Elbalal**: Conceptualization; project administration; supervision; validation. **Abdallah Abd AL‐Kareem Gibriel**: Supervision.

## CONFLICTS OF INTEREST

The authors declare no conflicts of interest.

## ETHICS STATEMENT

Ethical approval was obtained from center's ethical committee. Both verbal and written consents to publish this information were gained from the patients.

## TRANSPARENCY STATEMENT

The lead author Maali Mustafa affirms that this manuscript is an honest, accurate, and transparent account of the study being reported; that no important aspects of the study have been omitted; and that any discrepancies from the study as planned (and, if relevant, registered) have been explained.

## Data Availability

The data used and analyzed during this study are available from the authors on reasonable request.
